# Schistosomiasis Mansoni Manifesting as Multiple Colonic Polyps

**DOI:** 10.7759/cureus.11755

**Published:** 2020-11-28

**Authors:** Muhammed M Akhtar, Nasser ALJuhani, Donia Younus, Ashwag H ALSahafi, Ayman Abouhamda

**Affiliations:** 1 Department of Internal Medicine, East Jeddah Hospital, Jeddah, SAU; 2 Department of Internal Medicine and Endocrinology, East Jeddah Hospital, Jeddah, SAU; 3 Department of Internal Medicine and Hematology, East Jeddah Hospital, Jeddah, SAU; 4 Department of Internal Medicine and Gastroenterology, East Jeddah Hospital, Jeddah, SAU; 5 Independent Researcher, National Coalition of Independent Scholars, Jeddah, SAU

**Keywords:** schistosoma mansoni, schistosomiasis, bilharziasis, colonic polyps, pancytopenia, yemeni patient

## Abstract

Schistosomiasis (bilharziasis) is a common parasitic disease in subtropical and tropical parts of Africa, some parts of the Middle East, South America, Asia, and some parts of the Caribbean. It is a major public health problem and associated with significant morbidity and mortality in endemic areas. We describe a 28-year-old male patient presenting with bleeding per rectum associated with mucus secretion, abdominal pain, anorexia, and weight loss. Blood investigation showed pancytopenia with macrocytic hypochromic anemia. Meanwhile, his colonoscopy showed two large polyps 10 cm and 50 cm away from the anal verge, each measuring 3 cm in size. Microscopic examination of multiple colonic biopsies confirmed *Schistosoma mansoni*. The patient was treated with praziquantel, which improved his condition. Colonic schistosomiasis is an important differential diagnosis in patients with a history of travel to endemic areas. Early diagnosis and medical management can avoid unnecessary invasive intervention in such cases.

## Introduction

Schistosomiasis, also known as bilharzia, is an intravascular parasitic infection caused by schistosomes-intravascular parasitic trematodes belonging to the phylum platyhelminthes [1,2]. It has been officially named by the World Health Organization (WHO) as the third most devastating tropical disease, especially in developing countries [3]. *Schistosoma *has five main species that can infect humans: *Schistosoma mansoni*, *Schistosoma japonicum*, *Schistosoma mekongi*, *Schistosoma intercalatum*, and *Schistosoma haematobium *[3]. Moreover, *Schistosoma mansoni*, which is responsible for intestinal manifestations, as well as *Schistosoma haematobium*, which affects mainly the urinary bladder, are the most common types of schistosomiasis infecting humans [4]. Regarding mode of transmission, all the species are contracted the same way, by direct contact with infected water by the parasite cercariae*. *The parasite penetrates the host's skin sending eggs during its lifecycle to the intestinal wall and urinary bladder [3,5]. Clinically, *Schistosoma mansoni* infection can present with many symptoms, including abdominal pain, diarrhea, fever, intestinal polyps, leukocytosis, and hepatosplenomegaly [5]. These parasites travel through the body, reaching the heart and lungs and generally reaching the portal vein near the liver. Once in the liver, these parasites mature and are carried by the mesenteric vein or the venous plexus of the bladder [5]. We present an uncommon case of *Schistosoma mansoni *infection with colonic polyps, which presented in an atypical fashion compared to other reported colonic polyps *Schistosoma mansoni* cases in the literature.

## Case presentation

A 28 years old Yemeni male working as a house painter and living in Jeddah, Saudi Arabia, presented to the department of internal medicine in East Jeddah Hospital complaining of painless bleeding per rectum, which was associated with mucus, episodic abdominal pain, anorexia, and weight loss of four weeks duration. He denied any history of fever, nausea, vomiting, diarrhea, jaundice, headache, skin rash, joint pain, respiratory symptoms, mosquito bites, or swimming while his eating habits were considered unhealthy, eating from local and fast-food restaurants. His past medical and surgical history was unremarkable. Socially, the patient did not drink alcohol but often chewed tobacco and was of intermediate socioeconomic status. Upon further questioning, he reported a travel history to the Republic of Yemen 18 months before the beginning of his symptoms.

On physical examination, he was hemodynamically stable but was slightly underweight, with a body mass index of 18.3. There was no clinical evidence of pallor, jaundice, cyanosis, clubbing of fingers, dehydration, or lymphadenopathy. Furthermore, his abdominal examination was insignificant, with a soft abdomen with no tenderness, organomegaly, or ascites. However, his rectal examination showed bright blood mixed with mucus. Laboratory examination revealed a slightly low white blood cell (WBC) count of 2.35 x 10^3^ per microliter (μL), hemoglobin (Hb) 10.3 grams/deciliter (gm/dL) with a hematocrit of 28.8% accompanied by macrocytosis and hypochromia. His platelets were 71.5 x 10^3^/µL, while his coagulation profile, prothrombin time (PT), and partial thromboplastin time (PTT) were all normal. Regarding his liver profile, serum alanine aminotransferase (ALT) was 88 U/L, aspartate transaminase (AST), alkaline phosphatase (ALP), and serum protein and albumin were normal. As for the rest of the blood work, serum urea, creatinine, random blood glucose, and electrolytes were normal, and his serology panel was negative. As a result, examining a blood smear was opted, where it showed leukopenia with anemia and thrombocytopenia without any signs of blast cells. Afterward, stool analysis showed red and pus cells but did not reveal *Schistosoma *eggs, while his urine analysis was normal with no signs of infection (Table 1).

**Table 1:** Main hematological, biochemical, and serological data WBC: white blood cell, RBC: red blood cell, MCV: mean corpuscular volume, MCHC: mean corpuscular hemoglobin concentration, ALT: alanine aminotransferase, AST: aspartate aminotransferase, GGT: gamma-glutamyl transferase, IHA: indirect hemagglutination

Test	Direction	Result	Normal Range
WBC	↓	2.35 x 10^3^	4-11 x10^3^ per microliter (μL)
Neutrophils	↓	38.7 %	40-80%
Lymphocytes	-	39.7 %	20-45%
Eosinophils	↑	8.17 %	1-5%
RBC	↓	2.69 x 10^6^	4.5-6.5 x10^6^ cells/μL
Hemoglobin	↓	10.3	13-17 gram (gm)/ deciliter (dL)
Hematocrit	↓	28.8	40-50%
MCV	↑	107	76-96 fl/red cell
MCHC	↑	35.9	31.5-34.5 gm/dL
Platelets	↓	71.5 x 10^3^	150-400 x 10^3 ^/µL
Albumin	-	3.9	3.5-5 gm/dL
ALT	↑	88	5-55 U/L
AST	↑	37	5-34 U/L
GGT	↑	175	12-64 U/L
Total bilirubin	-	1.17	0.19-1.19 mg/dL
Direct bilirubin	↑	0.55	0-0.52 mg/dL
Creatinine	-	0.82	0.67-1.3 mg/dL
Serum vitamin B-12	↓	148	187-887 pg/ml
Schistosoma Antibodies (IHA)	↑	1:320	< 1:160

**Figure 1:**
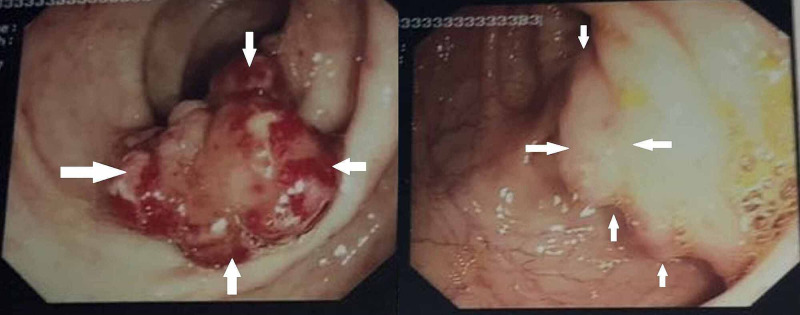
Colonoscopy showing two large polyps at 10 cm from the anal verge, each measuring 3 cm in diameter White arrows showing the margins of the polyps found during colonoscopy

Abdominal ultrasound revealed a slightly enlarged liver of 14.6 cm with mild surface irregularities that would suggest early signs of cirrhosis with no apparent focal lesions, while the spleen was of normal size of 12 cm with normal echogenicity. In addition, the portal vein diameter was normal. A decision to test the patient for *Schistosoma *and malaria were because of his travel history. Therefore, an indirect hemagglutination (IHA) kit assay for detection of *Schistosoma *antibody in the serum (Fumouze laboratories, France) was done and revealed a positive titer of 1:320 (cut-off titer of 1:160). Meanwhile, anti-parietal cell antibodies, anti-intrinsic factor antibodies, and malaria film all were negative. Consequently, colonoscopy was sought to determine the cause of bleeding, and the colonoscope was introduced up to the terminal ileum. Two large sessile polyps located 10 cm from the anal verge were seen, each measuring about 3 cm in size (Figure 1). A cross-section of colonic polyps as well as terminal ileum biopsies were taken.

**Figure 2:**
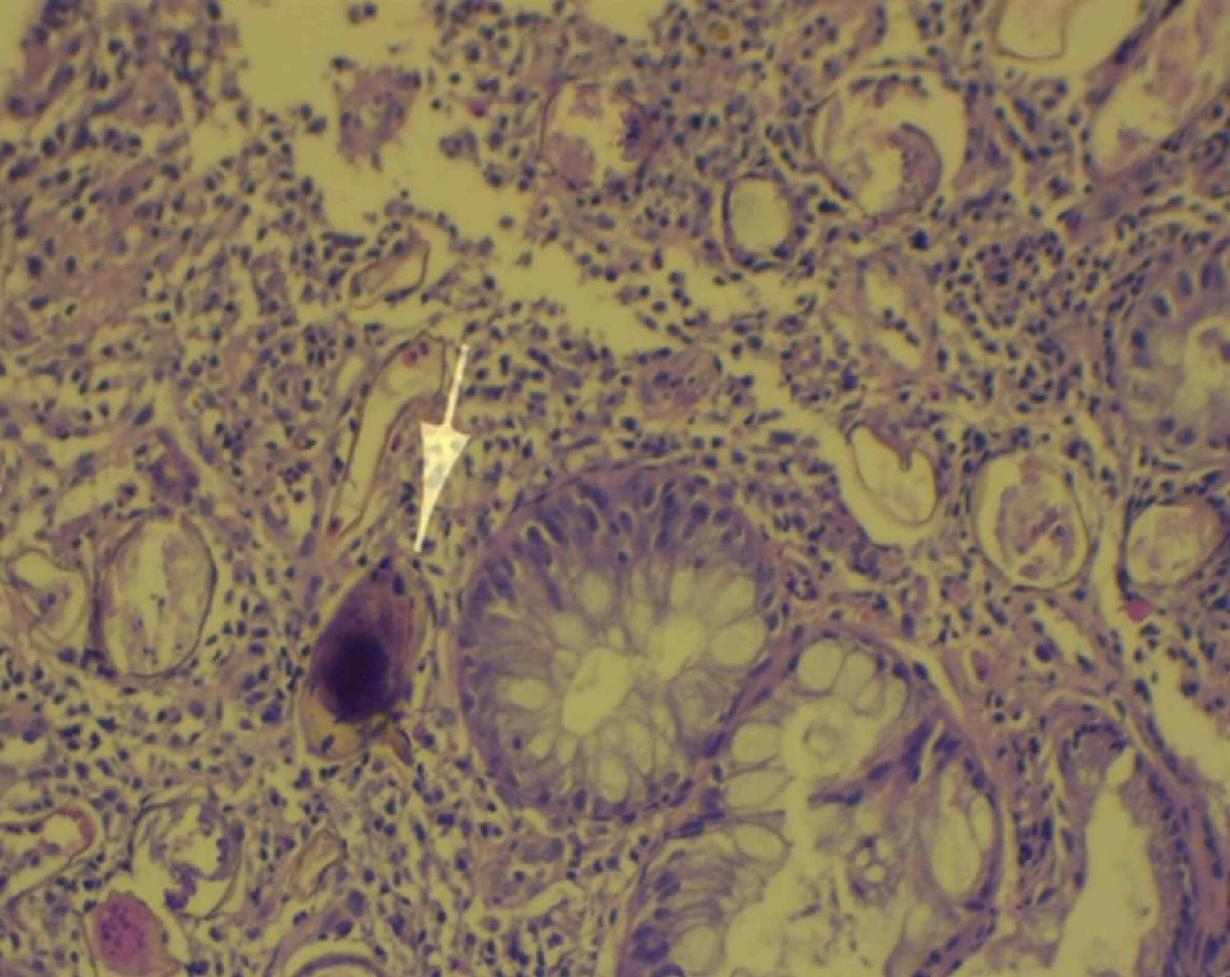
Histopathology of fragments of colonic polyps showed chronic inflammatory infiltrate with a sprinkle of eosinophils with characteristic Schistosoma mansoni eggs

Histopathological examination by staining the biopsy with hematoxylin and eosin (H&E) showed mild chronic inflammatory infiltrate with a sprinkle of eosinophils, which is a characteristic feature for *Schistosoma mansoni* eggs, with large prominent lateral spine near the posterior end with no evidence of malignancy (Figure 2). These findings confirmed the diagnosis of intestinal schistosomiasis associated with colonic polyps and possible hepatic *Schistosoma mansoni* infection. A decision to start the patient on praziquantel was recommended by the Internal Medicine Department with further follow-up plans for a colonoscopy, but the patient was lost to follow up.

## Discussion

Schistosomiasis manifestations are greatly variable in the gastrointestinal tract and are difficult to diagnose, as they often mimic many other pathologies [6]. While some of the parasitic eggs get left behind, they can lodge into the intestinal wall, colon wall, or even the liver and grow over the years slowly [7]. Furthermore, studies in countries with schistosomiasis endemics showed that intestinal and colon infections of the pathogen often infect young adults from 18 to 55 years old [8]. One of the most common features of all *S. mansoni *manifesting as a colon polyp is the chronicity, which is accompanied by at least one episode of fresh blood per rectum with recurrent abdominal pain and bloating [9,10]. Similarly, our patient was a young adult with chronic abdominal pain and bloody diarrhea and had a travel history in an endemic area where schistosomiasis is common. Not only that, he was from Yemen, where few reports state that it is often common for children to be infected with *S. mansoni* and *S. japonicum* and ​​​​present symptoms later in adulthood as intestinal manifestations and even colonic polyps [11]. Moreover, in rare incidents, a colonoscopy may be unhelpful in detecting the cause of the symptoms to be schistosomiasis*.* Alzahrani et al. reported a case of megacolon where colonoscopy showed no apparent or visible cause of the megacolon, and the patient had to undergo a laparotomy with extensive hemicolectomy only to discover by the histopathological report that the cause was schistosomiasis all along [12]. Therefore, when presented with abdominal manifestation of unknown etiology, it is best to emphasize and correlate the nationality, place of birth, travel history, symptoms, and age along with avoiding any invasive approach at first by having simple tests such as IHA and enzyme-linked immunosorbent assays (ELISAs), as both have been shown to have great sensitivity and specificity when combined [13], so missing such a crucial diagnosis would be avoided due to the similarities of the disease with other syndromes and pathogens.

## Conclusions

Colon polyps associated with schistosomiasis are rarely reported in non-endemic countries. However, patients presenting with background from places of known endemic areas, even if they spent years away from it or were born there, should raise a high index of suspicion to establish the correct diagnosis. History taking, pathogenic assays, and laboratory investigations can be great tools in detecting such rare incidents, such as our case, to eliminate any invasive interventions that might deem unnecessary later.
